# Direct reprogramming of fibroblasts into cardiomyocytes

**DOI:** 10.1186/s13287-017-0569-3

**Published:** 2017-05-25

**Authors:** Yueqiu Chen, Ziying Yang, Zhen-Ao Zhao, Zhenya Shen

**Affiliations:** 1 Institute for Cardiovascular Science & Department of Cardiovascular Surgery of The First Affiliated Hospital, Soochow University, 708 Renmin Road, Building 1, Room 1628, Suzhou, Jiangsu 215007 China; 2 Institute for Cardiovascular Science, Soochow University, 708 Renmin Road, Suzhou, Jiangsu 215007 China

**Keywords:** Direct reprogramming, Fibroblast, Cardiomyocyte, Transcription factor, MicroRNA, Small molecule

## Abstract

Cardiovascular diseases are the leading causes of death in the world. The limited regenerative capacity of adult cardiomyocytes is the major barrier for heart regeneration. After myocardial infarction, myofibroblasts are the dominant cell type in the infarct zone. Therefore, it is a good idea to reprogram terminally differentiated myofibroblasts into cardiomyocyte-like cells directly, providing a good strategy to simultaneously reduce scar tissue and increase functional cardiomyocytes. Transcription factors were first identified to reprogram myofibroblasts into cardiomyocytes. Thereafter, microRNAs and/or small molecules showed great potential to optimize the reprogramming process. Here, we systemically summarize and compare the major progress in directed cardiac reprogramming including transcription factors and miRNAs, especially the small molecules. Furthermore, we discuss the challenges needed to be overcome to apply this strategy clinically.

## Background

Cardiovascular disorders are the leading cause of human death in the world, and there were around 133 deaths per 100,000 people in 2013 according to the National Bureau of Statistics of China. This number was 136/100,000 in 2014. Over the past several decades, therapeutic approaches, including new drug development and cell transplantation, have had a limited effect on treating cardiovascular disorders in the clinic. Meanwhile, heart transplantation is restricted by the limited access to donor organs and itself has considerable mortality associated with immunosuppressant therapy and graft vasculopathy. Hence, it is important to explore novel therapeutic approaches for cardiac regenerative therapy.

The low regenerative capacity of cardiomyocytes is the main problem for heart repair. In order to get new cardiomyocytes, several strategies are developed, including: induction of pluripotent stem cells (iPSCs) and differentiation into cardiomyocytes [1, 2]; activation of cardiac stem cells or stimulation of cardiomyocytes to re-enter the cell cycle [3, 4]; and direct reprogramming of fibroblasts to cardiomyocytes [5, 6]. Cardiac fibroblast cells, accounting for up to 50% of all cardiac cells, survive well and couple with neighboring cells, and have been identified as an ideal cell source for direct reprogramming into cardiomyocytes [7]. After myocardial infarction, the fibroblasts expand and constitute the majority of the cells in the infarct zone [8, 9]. Therefore, reprogramming cardiac fibroblast cells into cardiomyocytes represents a promising and beneficial approach for cardiovascular regeneration.

*MyoD*, the master regulator gene for skeletal muscle cells, was discovered many years ago, but master regulators for other cell lineages were not been discovered until 2006 [10]. What is surprising is the innovative discovery that the transcription factors *Oct4*, *Sox2*, *Klf4*, and *c-myc* were capable of reprogramming terminally differentiated cells into iPSCs [11]. The induction of iPSCs provided new insights for direct reprogramming of adult cell types into specific lineages employing a combination of transcriptional factors [12], such as *Mgn3*, *Pdx1*, and *Mafa* for pancreatic β-cells [13], and *Gata4*, *Hand2*, *Mef2c*, and *Tbx5* for cardiomyocytes [14, 15]. Therefore, induction of cardiomyocytes from endogenous fibroblasts exhibits a feasible and promising approach to restore cardiac function following injury.

In this article, we review previous work on direct reprogramming of fibroblasts into cardiomyocytes using mouse and human cells, and discuss future efforts needed to apply this technique to the clinic.

### Direct cardiac reprogramming of murine fibroblasts

In 2010, postnatal cardiac and dermal fibroblasts were directly reprogrammed into cardiomyocyte-like cells in vitro, with a combination of three developmental transcription factors (GMT: *Gata4*, *Mef2c*, and *Tbx5*) by the group of Srivastava et al. [15] Thereafter, the same group demonstrated that retroviral delivery of GMT in vivo reprogrammed murine cardiac fibroblasts into induced cardiomyocytes, with decreased infarct size and modestly attenuated cardiac dysfunction [16]. Meanwhile, the group of Olson et al. reported increased reprogramming efficiency in vitro and in vivo by adding *Hand2* to the GMT combination, with improved cardiac function and reduced scar formation after myocardial infarction [14]. These studies provide a new insight into heart regeneration through gene therapy. Recently, non-integrated methods were developed to transfect mRNAs and proteins of cardiac transcription factors into fibroblasts to induce cardiomyocytes and cardiac progenitor cells, respectively [17, 18]. These reports provided safe methods for clinical application with great potential.

Based on the forced expression of cardiac transcription factors, many methods were developed to enhance the reprogramming efficiency, including inhibitor/cytokine treatments and epigenetic modulation [14, 16, 19]. SB431542 (a transforming growth factor (TGF)-β pathway inhibitor) can increase the conversion of both mouse embryonic fibroblasts and adult cardiac fibroblasts into cardiomyocyte-like cells up to fivefold based on the combination of *Gata4*, *Hand2*, *Mef2c*, *Tbx5*, and *Nkx2.5* [19]. Furthermore, inhibition of pro-fibrotic signaling (both TGF-β- and Rho-associated kinase pathways) reprograms embryonic fibroblasts into functional cardiomyocyte-like cells, with efficiency up to 60% for cTnT or α-actinin [20]. Besides inhibitors, cytokines, including fibroblast growth factor (FGF)2, FGF10, and vascular endothelial growth factor (VEGF), can increase the number of induced cardiac myocyte cells (iCMs) with spontaneous beating by 100-fold and accelerate the maturation of iCMs [21]. *Bmi1* acts as a critical barrier to iCM induction through epigenetic modulation, and reduced *Bmi1* expression changes the chromatin modification at cardiogenic loci, including increased active histone mark H3K4me3 and reduced repressive H2AK119ub. Correspondingly, cardiogenic gene expression was de-repressed during iCM conversion. These results indicate that the process of reprogramming is complex and influenced by many factors. Sequential addition of cytokines and inhibitors holds great promise for optimizing the protocol for cardiomyocyte reprogramming.

In addition to transcription factors and small molecules, microRNAs (miRNAs) have great influence on the expression of transcription factors such as *Gata4*, *Hand2*, *Mef2c*, T-box, and *Nkx2.5*, which regulate heart development. Thus, miRNAs represent an attractive and promising direction for reprogramming. miR-1 and miR-133 are cardiac and skeletal muscle-specific molecules, with miR-1 accounting for ~40% of miRNAs in the mammalian heart [22]. A “miRNA combo” (miR-1, miR-133, miR-208, and miR-499) was reported to convert cardiac fibroblasts into functional cardiomyocyte-like cells in vitro and in vivo [23, 24]. Adeno-associated virus (AAV) vectors are attractive tools for gene therapy, but the limited cargo size (~4.5 kb) of AAV restricts the expression of multiple transcription factors in one vector. However, considering the small size of miRNAs, it holds great potential to use miRNAs as gene therapy targets in vivo.

Above all, transcription factors and miRNAs play important roles during cardiac reprogramming [25–28]. Their functions in the cardiac development and direct reprogramming are summarized in Table 1.

**Table 1:** The functional mechanisms of transcription factors and microRNAs in the cardiac development and direct reprogramming

Factors	Direct reprogramming (crude, artificial transcription factor dosage)	Cardiac development (fine balance of transcription factor expression)
*GATA4*, *MEF2C*, *TBX5* [15]	*GATA4*, *MEF2C*, and *TBX5* are the core components of direct reprogramming	*NKX2.5*, *Mesp1*, and *Myocd*: expressed in cardiac progenitor cells (CPCs), and induce the development of cell fate to the mesoblastema layer *GATA4*, *HAND2*, and *TBX5*: induce the cardiac gene expression
*TBX5* [25]	Promotes the differentiation of transfected cells into beating cardiomyocytes	
*NKX2.5*	Induces *Ryr2* gene expression	
*Hand2*	Induces tropomyosin and cTnT in human dermal fibroblasts	
*Mesp1* [26]	Expressed in CPCs and programs nascent mesoderm toward a cardiovascular cell fate	
*Myocd*	Regulates the development of cardiomyocytes and smooth muscle cells, and increased the expression of cardiac sarcomeric proteins	
miR-1, miR-133, miR-208, miR-499 [24]	Alters H3K27 methyltransferase and demethylase expression	Promotes cardiomyocyte proliferation and suppresses apoptosis; increases expression of contractile proteins (MHC); influences the development of ventricular septum
miR-1 [27]	Promotes cardiomyocyte proliferation and suppresses apoptosis	Promotes cardiomyocyte proliferation and suppresses apoptosis
miR-133 [28]	miR-133-mediated *Snai1* repression	Promotes cardiomyocyte proliferation

During the iPSC induction process there is a pluripotent intermediate state, showing plastic developmental potential. After transfection of four Yamanaka factors and manipulating pathways for cardiogenesis, mouse embryonic fibroblasts (MEFs) can be reprogrammed into cardiomyocytes with spontaneous contraction [29]. Contracting cells resembling cardiomyocytes were also observed during iPSC induction via the chemical combination CRFVPTZ (CHIR99021, RepSox, Forskolin, VPA, Parnate, TTNPB, and DZnep). Furthermore, these chemically induced cardiomyocytes (CiCMs) are not generated through the iPSC stage, but via a cardiac precursor-like stage. These results indicate that the intermediate state is plastic and provides a new reprogramming strategy to generate cardiomyocytes [30]. Rgarding the safety problem of genetic manipulation, it is promising that the chemical cocktails could reprogram fibroblast cells into induced cardiomyocyte-like cells. However, considering reprogramming myocardial fibroblasts in situ, how to release these small molecules into the infarct region and reprogram myofibroblasts successfully into cardiomyocytes in vivo is still challenging. New materials that control drug release may overcome this problem. Meanwhile, it is worth noting that the strategy may have the risk of tumorigenicity since the specificity of small molecules cannot be guaranteed, and this procedure can also induce iPSCs. Typical methods for murine fibroblast reprogramming are summarized in Table 2.

**Table 2:** Factors and results in mouse direct cardiac reprogramming

Combination of factors	Original cell	Markers and efficiency	AP	Ca^2+^ transient	Beating
GMT [15]	CF, TTDF	cTnT^+^: 30% of α-MHC cells; α-actinin^+^: most of cTnT^+^ cells	+	+	+
OSKM; JI1, BMP4 [29]	MEF	cTnT^+^: 40%	+	+	+
GMT [16]	CF	α-MHC-EYFP^+^: ~40% at border zone	+	+	+
*miR-1*,*133*,*208*,*499*; JAK inhibitor I [23]	CF	α-MHC-GFP^+^: ~28%	+	+	+
GMT, *Myocd*, *Srf*, *Mesp1*, *Smarcd3* [42]	MEF	Myh6.Egfp^+^: 2.4%	–	+	–
*Hand2*, *Nkx2.5*, *Gata4*, *Mef2c*, *Tbx5* [43]	MEF, CF	Troponin T-GCaMP5^+^ activity: 1.6%	ND	+	+
OSKM; PEG hydrogel [44]	MEF, TTF	Beat patch per cm^2^: 9.4% α-actinin^+^: 1.72 fold/control	ND	+	+
GHMT, *MyoD* domain [45]	HF, LBF, TTF	cTnT^+^: 4.9%	ND	+	+
GHMT and SB431542 [19]	CF	Troponin T-GCaMP5^+^ activity: 9.27%	ND	+	–
GHMT, *Myod* domain [46]	HF	cTnT^+^: 19%	ND	+	+
GMT, *Mesp1*, *Myocd* and miR-133 [28]	MEF, CF	α-MHC-GFP & cTnT^+^: 8.1%; α-actinin^+^: 19.9%	ND		–
*OCT4*, SCPF [35]	MEF, TTF	beating clusters:~40/well of 24-well plate	+	+	+
GHMT [47]	MEF,	Sarcomere^+^: ~32%; NPPA^+^: 35% of sarcomere^+^; MYL2^+^: 22% of sarcomere^+^	+	ND	+
GMT mRNA, C_lipo [17]	CF	α-MHC-GFP^+^: 0.5% of transfected CF	ND	ND	–
*miR-1*, *miR-133*, *miR-208*, *miR-499* [24]	CF	tdTomato^+^ Troponin T^+^:12%	+	ND	+
OSKM, Ascorbic acid [48]	MEF	GATA4^+^: ~40%; MHC^+^: ~24%	+	ND	+
CHIR99021, RepSox, Forskolin, VPA [30]	MEF, TTF	α-actinin^+^: 14.5%; α-MHC^+^: 9%	+	+	+
GHMT, *miR-1*, *miR-133*, Y-27632, A83-01 [20]	MEF, AF	cTnT^+^: ~60% with A83-01; α-actinin^+^: ~60% with A83-01	+	+	+

A diverse range of factor combinations and original cells used in mouse cardiac reprogramming result in different efficiency, revealed by the expression of cardiomyocyte markers, electrophysiological characters, and beating property

GMT: *Gata4*, *Mef2c*, *Tbx5*; GHMT: *Gata4*, *Hand2*, *Mef2c*, *Tbx5*; OSKM: *Oct4*, *Sox2*, *Klf4*, *c-Myc*; SCPF: SB431542, CHIR99021, parnate, forskolin; Y-27632, Rock inhibitor; A83-01, TGF-β inhibitor

*AF* adult fibroblast, α*-MHC* α-myosin heavy chain, *AP* action potential, *CF* cardiac fibroblast, *cTnT* cardiac troponin T, *HF* head fibroblast, *LBF* low body fibroblast, *MEF* mouse embryonic fibroblast, *ND* not detected, *TTDF* tail-tip dermal fibroblast, *TTF* tail tip fibroblast

### Direct cardiac reprogramming of human fibroblasts

Compared to murine fibroblasts, it takes a long time to reprogram human fibroblasts into cardiomyocytes and it is more difficult to obtain mature cardiomyocytes from human somatic cells. After reprogramming in mice, Nam et al. [31] reported in 2013 that the combination of *GATA4*, *HAND2*, *TBX5*, *MYOCD* (myocardin), miR-1, and miR-133 activated cardiac marker expression, but that most induced cardiomyocytes were in a partially reprogrammed state. In the same year, Wada et al. [32] discovered that reprogramming fibroblasts with the transcription factors *GATA4*, *MEF2C*, *TBX5*, *MESP1*, and *MYOCD* (referred to as GMTMM) changed the cell morphology from a spindle shape to a rod-like shape, and exhibited spontaneous Ca^2+^ oscillations. Srivastava and colleagues discovered that GMT (*GATA4*, *MEF2C*, and *TBX5*) was insufficient for reprogramming of human fibroblasts into cardiomyocytes, and the addition of *ESRRG* and *MESP1* to GMT could induce cardiomyocyte-like cells with cardiac-specific gene expression and sarcomere formation. Furthermore, the addition of *MYOCD* and *ZFPM2* resulted in more features of cardiomyocytes, including global cardiac gene expression and a phenotypic shift to a cardiac state [6]. Although the reprogramming efficiency in human cells is very low, these reports represent a great step towards therapeutic application in the clinic.

The abovementioned three reports in human cells all concern transcription factors delivered through a virus until Ding et al. reported on small molecules [36]. Small molecules have effects in the reprogramming of human pancreatic lineages and neural stem cells from somatic cells [33, 34], and also have enormous influence in the process of transdifferentiation of fibroblasts toward cardiomyocytes with reduced transcription factor numbers [35]. Thereafter, Ding’s group found that human somatic cells could be transdifferentiated to cardiomyocyte-like cells which resembled naive human cardiomyocytes with regards to the properties of transcriptome, epigenetics, and electrophysiology, with nine small molecules (9C: CHIR99021, A83-01, BIX01294, AS8351, SC1, Y27632, OAC2, SU16F, and JNJ10198409) in 2016 [36]. Moreover, human fibroblasts treated with 9C could be converted into cardiomyocytes in the infarcted mouse heart, and enhanced the function of infarcted heart [36]. To understand cardiac reprogramming further, we summarized typical methods for human fibroblast reprogramming (Table 3).

**Table 3:** Factors and results in direct cardiac reprogramming of human cells

Factors	Original cell	Markers and efficiency	AP	Ca^2+^ transient	Beating
*ETS2*, *MESP1* [49]	DF	NKX2.5-tdTomato^+^: 30 colonies/plate (cardiac progenitor)	–	–	–
*GATA4*, *MEF2C*, *TBX5*, *MESP1*, *MYOCD* [32]	HCF	cTnT^+^: 5.9% α-actinin^+^: 5.5%	+	+	+
*GATA4*, *MEF2C*, *TBX5*, *ESRRG*, *MESP1*, *MYOCD*, *ZFPM2* [6]	ESC, FH, neonatal skin	α-MHC-mCherry^+^: 15.8% α-MHC-mCherry^+^ & cTnT^+^: 13%	+	+	ND
*GATA4*, *MEF2C*, *TBX5*, *MESP1*, *MYOCD*, *miR-133* [28]	HCF	cTnT^+^: 27.8% α-actinin^+^: 8%	ND	+	+
*GATA4*, *HAND2*, *MYOCD*, *TBX5*, *miR-1*, *miR-133* [31]	HFF	cTnT^+^: 34.1%	ND	+	+
CHIR99021, A83-01, BIXO1294, AS8351, SC1, Y27632, OAC2, SU16F, JNJ [36]	HFF	cTnT^+^: 6.6%	+	+	+

Differential factors combination and original cells used in human cardiac reprogramming result in different efficiency, revealed by the expression of cardiomyocyte markers, electrophysiological characters and beating property

α*-MHC* α-myosin heavy chain, *AP* action potential, *cTnT* cardiac troponin T, *DF* dermal fibroblast, *ESC* embryonic stem cell, *FH* fetal heart, *HCF* human cardiac fibroblast, *HFF* human foreskin fibroblast, *ND* not detected

### Direct cardiac reprogramming in vivo

Reprogramming fibroblasts into cardiomyocytes in vivo is required for heart regeneration. Transplanting reprogrammed cells and transdifferentiation factors into the infarcted heart represent two strategies towards this purpose. Firstly, cardiac fibroblasts were transduced with GMT for 1 day and were transplanted into mouse hearts. These cells were reprogrammed to cardiomyocytes in vivo [15]. Thereafter, in situ repair of the heart was performed by targeting endogenous cardiac fibroblasts through virus transfection. After coronary ligation, resident non-myocytes in the infarct zone can be reprogrammed into cardiomyocyte-like cells by local delivery of GMT through a virus. In addition, thymosin β4 can improve the migration ability of fibroblasts. Co-injection of thymosin β4 and GMT further improved the ejection fraction and reduced scar formation [16]. Using a retrovirus expression system, forced expression of GHMT (GATA4, HAND2, MEF2C, and TBX5) can also reprogram cardiac fibroblasts into beating cardiomyocytes in vivo [14]. In fact, the cardiac niche in vivo improves the efficiency of transdifferentiation. This gives more hope to increasing the reprogramming efficiency and maturity [37]. These results suggest the possibility for repairing the heart through gene therapy by targeting myofibroblasts. However, a relatively safe gene delivery method needs to be developed. AAV vectors show great potential for gene therapy, but limited capacity restricts their application for multiple genes. Reprogramming with miRNAs may solve the problem; furthermore, cell-penetrating proteins also hold great promise.

Transplanting of human fibroblasts treated with 9C can efficiently get chemically induced cardiomyocytes in vivo and enhance the function of the infarcted heart [36]. Compared to transcription factors and miRNAs, small molecules have many advantages in vitro, such as better temporal control, more effective cell delivery, and they are non-immune, less expensive, and safer. Moreover, it is more convenient to control the process of programming through varying small molecule concentrations and combinations. However, there are still some questions about the use of small molecules for reprogramming in situ. Small molecules can enter the blood and spread to other organs with ambiguous influence, and the impact time should be strictly controlled to convert fibroblasts into target cells. Therefore, novel biomaterials should be developed to help local delivery of multiple drugs in a controllable manner.

## Conclusion

Although transcription factors, miRNAs, and small molecules have been proved important for reprogramming fibroblasts into cardiomyocytes, their reproducibility in different laboratories is low and the induced cardiomyocytes show different properties and maturity. There are several reasons for this instability. The different original cells have different tendencies for transdifferentiation into cardiomyocytes. Compared to tail tip fibroblasts, cardiac fibroblasts have more potential to be reprogrammed into cardiomyocytes [7]. The combination of different transcription factors may also influence the process of reprogramming. In addition, the induction medium and the time spent in the process of induction have important roles during reprogramming. Moreover, different criteria give rise to different success levels in the process, and thus a detailed standard is needed to define the level of induced cardiomyocytes, besides spontaneous beating and being calcium transient. Thus, optimization of the minimal and effective combination to improve reprogramming efficiency is required, including the epigenetic status, maturation, and the integration ability into the infarcted heart. Overall, direct reprogramming is a complex process influenced by many factors, and there are still many issues to be resolved.

Compared with iPSC preparation and cardiac differentiation, direct reprogramming may eliminate the risk of teratoma formation and shorten the time for cell transplantation. There are many differences in the cellular, molecular, and electrophysiological levels of the de novo cardiomyocytes induced from iPSCs and direct reprogramming [20, 38, 39] (Table 4). In addition, the iCMs from in situ direct reprogramming may have better interaction with cells in the heart [40]; this may reduce the risk of arrhythmias compared to cell transplantation. Furthermore, considering myofibroblasts as the dominant cell type in the infarct zone, direct reprogramming may reduce the scar size. Local delivery of gene therapy vectors or small molecules holds great promise for heart regeneration.

**Table 4:** The differences of cardiomyocytes induced from iPSCs and direct reprogramming

Properties	iPSC/hPSC-derived cardiomyocytes	Direct reprogrammed cardiomyocytes	Adult cardiomyocytes
Differentiation efficiency	>80%	~60% cTnT^+^ α-actinin^+^ [20]	/
Size	Small size (membrane capacitance 18 pF) [38], 1/10 of physical size of adult cardiomyocytes	Small size	Membrane capacitance 150 pF
Nucleus	Mono-nuclear [39]	Mono-nuclear	Bi- or multi-nuclear
Morphology	Circular or irregular shape	Spindle-shape	Rod-shape
Sarcomere	Better organized	Disarrayed	Highly organized
Primary metabolic substrate	Glucose	Glucose	Fatty acid
Markers	α-MHC^+^, α-actinin^+^, Troponin T^+^	α-MHC^+^, α-actinin^+^, Troponin T^+^	α-MHC^+^, α-actinin^+^, Troponin T^+^
Ca^2+^ transient	+	+ (few induced cardiomyocytes)	+
Electrophysiology	Resting membrane potential –60 mV (slower action potential)	Resting membrane potential –48 mV (slowest action potential)	Resting membrane potential –90 mV (quicker action potential)

*hPSC* human pluripotent stem cell, *iPSC* induce pluripotent stem cell, / no data

**Fig. 1:**
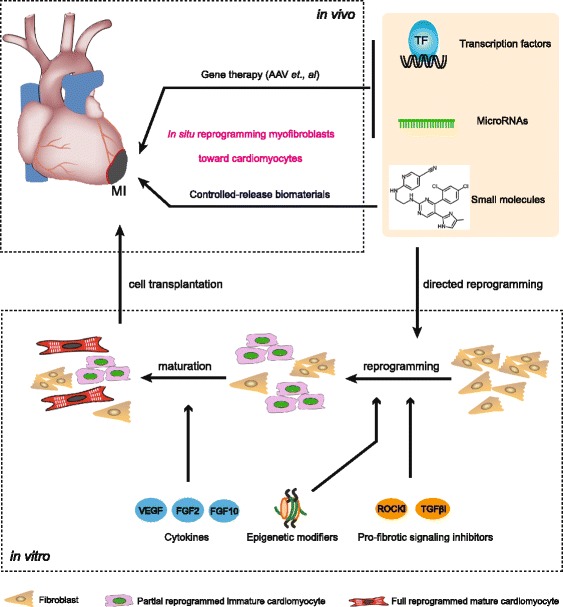
The potential application of reprogramming fibroblasts into cardiomyocytes. The means of direct reprogramming through transcription factors, microRNAs, and small molecules are shown in vitro and in vivo. *AAV* adeno-associated virus, *FGF* fibroblast growth factor, *MI* myocardial infarction, *TGF* transforming growth factor, *VEGF* vascular endothelial growth factor

In this review, we have discussed the potential application of reprogramming fibroblasts into cardiomyocytes (Fig. 1). Over the past years, direct reprogramming in the heart has made significant progress and has important implications in understanding the biology of heart development. All studies in cardiac cell reprogramming have been positive in mouse cardiovascular disease models, but much more remains to be done to overcome the challenges during clinical translation. One of the major challenges in the field of direct cardiac reprogramming is the low efficiency [25]. Another challenge is the heterogeneity which is demonstrated by high-resolution single-cell sequencing [41]. Patch clamp also confirmed the presence of pacemaker, ventricular, and atrial cardiomyocytes. Therefore, the potential risk of arrhythmias still exists because of the different electrophysiology properties. However, this risk is ignored in the mouse because its heart rate is quite different from humans. Therefore, it will also necessary to conduct large animal studies, such as in pigs and monkeys, to verify the safety of directed reprogramming.

Above all, following the discoveries of key transcription factors, miRNAs, cytokines, small molecules, gene delivery methods, and novel biomaterials, reprogramming mechanisms will be clarified. After systematic research in large animals, directed cardiac reprogramming may ultimately contribute to heart repair.

## Abbreviations

AAV: Adeno-associated virus
Bmi 1: BMI1 proto-oncogene
CiCM: Chemically induced cardiomyocyte
c-myc: v-myc avian myelocytomatosis viral oncogene homolog
cTnT: Troponin T2
FGF: Fibroblast growth factor
Gata4: GATA binding protein 4
Hand2: Heart and neural crest derivatives expressed transcript 2
iCM: Induced cardiac myocyte cell
iPSC: Induced pluripotent stem cell
Mafa: v-maf musculoaponeurotic fibrosarcoma oncogene family, protein A
MEF: Mouse embryonic fibroblast
Mef2c: Myocyte enhancer factor 2C
MESP1: Mesoderm posterior 1
MyoCD: Myocardin
MyoD: Myogenic differentiation 1
NKX2.5: NK2 homeobox 5
OCT4: Organic cation/carnitine transporter4
Pdx1: Pancreatic and duodenal homeobox 1
SOX2: SRY (sex determining region Y)-box 2
Tbx5: T-box 5
TGF: Transforming growth factor
α-actinin: Alpha-actinin protein
